# First-Line Management of Advanced High-Grade Serous Ovarian Cancer

**DOI:** 10.1007/s11912-020-00933-8

**Published:** 2020-06-04

**Authors:** Reem D. Mahmood, Robert D. Morgan, Richard J. Edmondson, Andrew R. Clamp, Gordon C. Jayson

**Affiliations:** 1 Department of Medical Oncology, Christie NHS Foundation Trust, Wilmslow Road, Withington, Manchester, M20 4BX UK; 2 Faculty of Biology, Medicine and Health, University of Manchester, Manchester, UK; 3 Department of Gynaecological Oncology Surgery, Saint Mary’s Hospital, Manchester University NHS Foundation Trust, Oxford Road, Manchester, UK

**Keywords:** Ovarian cancer, Bevacizumab, PARP inhibitors, Cytoreductive surgery, Intra-peritoneal chemotherapy

## Abstract

**Purpose of Review:**

Epithelial ovarian cancer is a disease that encompasses a number of histologically and molecularly distinct entities; the most prevalent subtype being high-grade serous (HGS) carcinoma. Standard first-line treatment of advanced HGS carcinoma includes cytoreductive surgery plus intravenous paclitaxel/platinum-based chemotherapy. Despite excellent responses to initial treatment, the majority of patients develop recurrent disease within 3 years. The introduction of the vascular endothelial growth factor (VEGF) inhibitor, bevacizumab, and poly(ADP-ribose) polymerase (PARP) inhibitors into first-line management has changed the outlook for this lethal disease. In this review, we summarise the most recent clinical trials that determine current primary therapy of advanced HGS carcinoma and the ongoing trials that aim to change management in the future.

**Recent Findings:**

Recent phase III clinical trials have shown that delayed primary surgery after completing neo-adjuvant chemotherapy is non-inferior to immediate primary surgery, but could provide a survival benefit in FIGO (International Federation of Gynecology and Obstetrics) stage IV disease. The use of weekly intravenous chemotherapy regimens has not been proven to be more effective than standard 3-weekly regimens in Western patient populations, and the use of intraperitoneal chemotherapy remains controversial in the first-line setting. In contrast, newer systemic anti-cancer therapies targeting angiogenesis and/or HR-deficient tumours have been successfully incorporated into front-line therapeutic regimens to treat HGS carcinoma. Recent results from randomised trials investigating the use of PARP inhibitors as monotherapy and in combination with the anti-angiogenic agent, bevacizumab, have demonstrated highly impressive efficacy when combined with traditional first-line multi-modality therapy.

**Summary:**

Management of HGS carcinoma is evolving, but further work is still required to optimise and integrate tumour and plasma biomarkers to exploit the potential of these highly efficacious targeted agents.

## Introduction

Ovarian cancer is the 8th commonest cancer diagnosed in women and the 8th commonest cause of female cancer-related death worldwide [1]. Approximately 300,000 women were diagnosed with ovarian cancer in 2018 [1]; the majority being diagnosed with FIGO (International Federation of Gynecology and Obstetrics) stage III/IV disease [2, 3]. Ovarian cancer originates from the epithelial cells (ovarian surface/distal fallopian tube epithelium) in around 90% of cases, with the histological subtype high-grade serous (HGS) accounting for 70% of cases [4]. Standard first-line treatment of advanced HGS carcinoma involves cytoreductive (debulking) surgery with paclitaxel/platinum-based chemotherapy [5, 6]. Despite excellent initial treatment responses in around 70% of women, the majority of patients develop recurrent disease within 3 years of their primary therapy [7].

First-line treatment for advanced stage HGS carcinoma provides the only realistic opportunity for cure, with a 5-year survival of 27% in patients with FIGO stage III disease [8]. In women whose disease is not cured, first-line treatment often leads to the longest disease-free interval (DFI), allowing for the greatest time period to regain quality of life during the disease. For these reasons, research has focused on therapeutic strategies that optimise outcomes of primary therapy. This review summarises the key clinical trial data that have led to the current status of first-line treatment of advanced HGS carcinoma.

## Primary Cytoreductive Surgery

Primary cytoreductive surgery allows for accurate FIGO staging and should be directed towards achieving complete debulking (i.e., no residual disease [RD]) [9, 10]. Traditionally, immediate primary surgery (IPS) was performed followed by adjuvant platinum-based chemotherapy. However, a proportion of women diagnosed with advanced stage disease are unable to undergo IPS due to contra-indications, including comorbidities and/or disease-related factors e.g. tumour burden [11]. For these patients, delayed primary surgery (DPS) after completing 3–4 cycles of platinum-based neoadjuvant chemotherapy (NACT) was demonstrated as a non-inferior therapeutic option in two randomised phase III trials by comparison of the primary outcome of overall survival (OS) [12, 13]. In the EORTC 55791 trial, 632 women diagnosed with FIGO stage IIIC-IV epithelial ovarian cancer (EOC) (61.8% serous adenocarcinoma) were randomised to undergo either IPS followed by at least 6 cycles of adjuvant platinum-based chemotherapy versus 3 cycles of platinum-based NACT followed by DPS and a further 3 (or more) cycles of adjuvant chemotherapy [12]. In the CHORUS trial, 550 women diagnosed with FIGO stage IIIA-IV EOC (70.5% HGS) were randomised to undergo IPS followed by 6 cycles of adjuvant platinum-based chemotherapy or 3 cycles of platinum-based NACT followed by DPS and a subsequent 3 cycles of chemotherapy [13]. There was no difference in overall survival (OS) between groups in either trial (EORTC 55791: HR 0.98, 95% CI 0.84–1.13; CHORUS: HR 0.87 95% CI 0.72–1.05). However, in a subsequent meta-analysis including individual patient data from both trials, patients diagnosed with FIGO 1988 stage IV disease had significantly improved OS (24.3 versus 21.2 months, HR 0.76, 95% CI 0.58–1.00, *p* = 0.048) if they were treated with NACT followed by DPS [14•]. Both EORTC 55791 and CHORUS demonstrated that peri- and post-operative morbidity/mortality occurred less frequently in those women undergoing NACT-DPS [12, 13].

EORTC 55971 and CHORUS have been criticised due to the low number of patients in whom surgery achieved complete cytoreduction at IPS; debulking to less than 1 cm of residual disease was achieved in 42.3% of patients in EORTC 55971 and 41.6% of patients in CHORUS [12, 13]. As a result, TRUST (NCT02828618) was initiated, which is an ongoing randomised phase III trial recruiting women diagnosed with FIGO stage IIIB-IVB EOC within quality-assured gynaecological oncology centres that fulfil certain criteria including that they carry out more than 36 debulking surgeries a year, achieve complete cytoreduction in ≥ 50% of cases and are willing to undergo audits from the TRUST committee to verify these numbers [15]. Nevertheless, EORTC 55971 and CHORUS led to the paradigm shift in clinical practice, in which DPS is now routinely considered in patients unlikely to achieve no RD/complete cytoreduction with IPS and those with comorbidities indicating high peri-operative morbidity/mortality risk [5, 6]. Factors which impact on the ability to achieve complete cytoreduction include extra-abdominal metastases, extensive bowel involvement or complex blood vessel involvement [16, 17].

During primary cytoreductive surgery, the removal of bulky or suspicious lymph nodes is part of complete cytoreduction. However, complete pelvic lymph node resection at time of surgery is not currently recommended [6]. Retrospective analysis had suggested an OS benefit with complete systematic pelvic and para-aortic lymphadenectomy [18, 19]. A phase III trial then showed that systematic resection of pelvic and para-aortic lymph nodes compared to resection of bulky lymph nodes only did not provide a survival benefit. But this trial was criticised due to the inclusion of patients with complete resection and residual disease up to 1 cm after surgery [20]. The phase III LION trial was set up to determine if systematic lymph node dissection prolongs OS with a more homogeneous group of patients [21•]. Women (*n* = 647) were recruited with diagnosed FIGO stage IIB-IV EOC and were randomly allocated intraoperatively to proceed to complete lymphadenectomy or not if they met the following criteria: achieved complete macroscopic resection, intraoperatively remained in good condition and had no clinically positive lymph nodes. The study showed that systematic lymphadenectomy increased detection of sub-clinical retroperitoneal metastases in 56% of patients but was not associated with improved OS (69.2 versus 65.5 months, HR 1.06, 95% CI 0.83–1.34). Systematic lymphadenectomy did increase post-operative morbidity and mortality [21•]. The results from the LION trial confirm guidelines from the European Society of Medical Oncology (ESMO) that lymph node resection at primary surgery should focus only on nodes that are suspected to harbour metastatic disease [6].

## Dose-Dense First-Line Chemotherapy

Standard first-line chemotherapy for EOC includes 3-weekly platinum and 3-weekly paclitaxel chemotherapy [5, 6]. In the United Kingdom (UK), guidance from the National Institute for Health and Care Excellence (NICE) also currently recommends to offer single-agent platinum-based chemotherapy as an alternative to combination regimens [22]. Dose-dense regimens involve weekly administration of chemotherapy. Indeed, preclinical data showed that metronomic scheduling of docetaxel or paclitaxel was effective [23], and early phase trial data supported the evaluation of dose-dense taxanes within randomised phase III trials [24–27]. Four randomised phase III trials have been reported investigating dose-dense first-line therapies (Table 1) [28–31].

**Table 1:** PFS outcomes from first-line, phase III dose-dense chemotherapy trials [28–31]

Trial	PFS (months)		
	3-weekly carboplatin and 3-weekly paclitaxel	3-weekly carboplatin and weekly paclitaxel	Weekly carboplatin and weekly paclitaxel
JGOG3016	17.5	28.2 (HR 0.76, 95% CI 0.62–0.91, *p* = 0.0037)	N/A
MITO-7	17.3	N/A	18.3 (HR 0.96, 95% CI 0.80–1.16, *p* = 0.66)
ICON 8	17.7	20.8 (*p* = 0.35)	21.0 (*p* = 0.51)
GOG 262 + 3-weekly bevacizumab	14.0	14.7 (HR 0.89, 95% CI 0.74–1.06, *p* = 0.18)	N/A

The initial JGOG trial showed an advantage to dose dense chemotherapy but this was not confirmed in subsequent randomised trials (MITO-7, ICON8 and GOG 262). In addition, currently ongoing is the ICON 8B trial which is comparing weekly chemotherapy regimens with 3-weekly regimens, with concurrent bevacizumab (7.5 mg/kg) 3-weekly for a maximum of 22 cycles. The primary outcomes of ICON8B are PFS and OS

In JGOG 03016, 631 women diagnosed with FIGO stage II–IV EOC (55.7% serous adenocarcinoma) were recruited from 85 centres in Japan. Women were randomised to 3-weekly carboplatin (AUC6) and weekly paclitaxel (80 mg/m^2^) versus 3-weekly carboplatin (AUC6) plus 3-weekly paclitaxel (180 mg/m^2^) [28]. JGOG 03016 reported a significant improvement in PFS (28.0 versus 17.2 months, HR 0.71, 95% CI 0.58–0.88) and OS (100.5 versus 62.2 months, HR 0.79, 95% CI 0.63–0.99, *p* = 0.39) in the dose-dense weekly paclitaxel arm [28, 32]. Similar toxicity profiles were reported in each treatment arm, although the prevalence of myelosuppression was greater in the dose-dense arm and negatively impacted on the ability to complete the target 6 cycles of chemotherapy (Table 2) [28]. The results from this trial were encouraging, although additional data was required to determine if the trends were reproducible within different ethnic populations.

**Table 2:** Toxicities reported in dose-dense chemotherapy trials [28–31]

Trial	JGOG3016		MITO-7		ICON8			GOG 262	
	3-weekly carboplatin and 3-weekly paclitaxel	3-weekly carboplatin and weekly paclitaxel	3-weekly carboplatin and 3-weekly paclitaxel	Weekly carboplatin and weekly paclitaxel	3-weekly carboplatin and 3-weekly paclitaxel	3-weekly carboplatin and weekly paclitaxel	Weekly carboplatin and weekly paclitaxel	3-weekly carboplatin and 3-weekly paclitaxel ± 3-weekly bevacizumab	3-weekly carboplatin and weekly paclitaxel ± 3-weekly bevacizumab
Toxicity									
Grade 3–4 anaemia	44%	69%*	8%	6%	5%	13%*	5%	16%	36%*
Grade 3–4 neutropenia	88%	92%	50%	42%*	15%	35%	30%	83%	72%*
Grade 3–4 thrombocytopenia	38%	44%	7%	1%*	3%	8%	2%	16%	20%
Grade 2 or higher neuropathy	6%	7%	17%	6%*	27%	24%	22%	18%	26%*

*A statistically significant difference (*p* < 0.05)

Three randomised phase III trials have since been reported investigating dose-dense therapy in European (MITO-7, ICON8) and North American (GOG 262) populations [29–31] (Table 1). In MITO-7, 822 women, from Italy or France, diagnosed with FIGO stage IC-IV EOC (69.6% serous adenocarcinoma) were randomly allocated to receive either 3-weekly carboplatin (AUC6) plus 3-weekly paclitaxel (175 mg/m^2^) or weekly carboplatin (AUC2) plus weekly paclitaxel (60 mg/m^2^) [29]. In ICON8, 1566 women, from the UK, Ireland, Australia, New Zealand, Mexico or South Korea, diagnosed with FIGO stage IC-IV EOC (68.5% HGS) were randomised to receive either 3-weekly carboplatin (AUC5/6) plus 3-weekly paclitaxel (175 mg/m^2^) or one of two experimental arms: 3-weekly carboplatin (AUC5/6) plus weekly paclitaxel (80 mg/m^2^) or weekly carboplatin (AUC2) plus weekly paclitaxel (80 mg/m^2^) [30••]. Neither MITO-7 nor ICON8 showed an improvement in PFS in the dose-dense experimental arms [29, 30] (Table 1).

In GOG 262, 692 North American women diagnosed with FIGO III-IV incompletely resected at IPS or FIGO II–III with residual lesions ≤ 1 cm after IPS EOC (88% serous adenocarcinoma) were randomised to receive 3-weekly carboplatin (AUC6) with either 3-weekly paclitaxel (175 mg/m^2^) or weekly paclitaxel (80 mg/m^2^) [31]. In addition, all patients were given the option of incorporating the anti-angiogenic agent, bevacizumab (15 mg/kg; 3-weekly from cycle 2 until disease progression or intolerable adverse event). In total, 84% of patients recruited to the trial chose to receive bevacizumab. In keeping with MITO-7 and ICON8, GOG 262 showed no significant difference in PFS between arms (14.7 versus 14.0 months, HR 0.89, 95% CI 0.74–1.06) [31].

Tolerability of weekly dose-dense chemotherapy differed between MITO-7, ICON8 and GOG 262 (Table 2) in that fewer patients who received weekly treatment had grade 3 or more neutropenia in MITO-7 and GOG 262, whereas the opposite was reported in ICON8. Moreover, the prevalence of grade 2 or more neuropathy was lower in patients receiving weekly treatment in MITO-7, higher in GOG 262, but not different in ICON8. The dose-dense MITO-7 regimen, which incorporated a lower dose of weekly paclitaxel to the other studies (60 mg/m^2^ versus 80 mg/m^2^), was tolerated better by patients, and patients reported significantly better quality of life scores than those on 3-weekly treatment. This could justify the use of this regimen in patients with advanced stage disease and poorer performance status (i.e. 3 to 4). Weekly carboplatin (AUC2) plus paclitaxel (60 mg/m^2^) is recommended by the National Comprehensive Cancer Network (NCCN) for patients with a poorer ECOG (Eastern Cooperative Oncology Group) performance status or for use in patients > 70 years old and/or those with comorbidities (e.g. renal disease) [5]. However, all trials limited inclusion criteria to those with an ECOG performance status of 2 or less, so tolerability of weekly regimens may not be translated clinically to other subgroups of patients [33].

Although the reason for differences in efficacy between trials recruiting East Asian or European/North American women is unknown, it may be explained through pharmacogenetics [34, 35]. In addition, the interaction between bevacizumab and dose-dense regimens remains unclear and will be addressed in the ongoing ICON8B trial. Due to the variation in trial results, at present, the use of dose-dense chemotherapy is not considered standard of care management for the first-line treatment of advanced HGS cancer in Western populations.

## Intraperitoneal and Hyperthermic Intraperitoneal Chemotherapy

Intraperitoneal (IP) chemotherapy delivers greater concentrations of cytotoxic agents to the peritoneal cavity compared to intravenous (IV) chemotherapy [36]. The NCCN guidelines suggest IP chemotherapy as a potential option for FIGO stage II–III EOC following optimal debulking (< 1 cm RD) [5]. This guidance was based on improved survival outcomes reported in the randomised phase III trial, GOG 172 [37]. ESMO guidelines recognise this trial but still regard IP chemotherapy as experimental and difficult to administer [6].

In GOG 172, women (*n* = 429 women) that were optimally debulked at IPS (< 1 cm RD), with FIGO stage III EOC (79% serous adenocarcinoma), were randomised to receive 6 cycles of 3-weekly IV paclitaxel (135 mg/m^2^ on day 1) plus IV cisplatin (75 mg/m^2^ on day 2) or 6 cycles of 3-weekly IV paclitaxel (135 mg/m^2^ on day 1) plus IP cisplatin (100 mg/m^2^ on day 2) and IP paclitaxel (60 mg/m^2^ on day 8). GOG 172 demonstrated that patients receiving IP chemotherapy had significantly improved PFS (23.8 versus 18.3 months, HR 0.80, 95% CI 0.64–1.00, *p* = 0.05) and OS (65.6 versus 49.7 months; HR 0.75, 95% CI 0.58–0.97, *p* = 0.03) [37]. Nevertheless, grade 3 and 4 adverse effects (e.g. increased pain, myelosuppression and gastrointestinal and neurological toxicity) occurred more frequently in patients treated with IP chemotherapy. In addition, the IP regimen was more frequently curtailed before completion of primary therapy, with only 42% of women receiving all 6 cycles [37]. The primary reasons for discontinuation were catheter-related e.g. infection and blockage [38]. Furthermore, post-publication discussion raised the question of the tolerability of the control arm, as only 83% of patients received all 6 cycles of IV chemotherapy, which were fewer patients than would be expected through treatment with carboplatin and paclitaxel. Aiming to address these criticisms and with the incorporation of maintenance therapies, GOG 252 was carried out.

In the randomised phase III trial, GOG 252, IP chemotherapy was delivered with concurrent IV bevacizumab [39•]. Here, women (*n* = 1560) diagnosed with FIGO stage II–IV EOC (72.2% HGS), who had undergone maximal cytoreduction at IPS, were randomised to receive IV weekly paclitaxel (80 mg/m^2^) plus one of three regimens: 3-weekly IV carboplatin (AUC6 on day 1); 3-weekly IP carboplatin (AUC6 on day 1) or 3-weekly IP cisplatin (75 mg/m^2^ on day 2) and 3-weekly IP paclitaxel (60 mg/m^2^ on day 8). All arms were given with the addition of 3-weekly bevacizumab (15 mg/kg on day 1 from cycle 2 for a maximum of 22 cycles). This trial did not report a significant difference in progression-free survival between arms (24.9 versus 27.4 versus 26.2 months, respectively) [39•]. The conflicting outcomes from these clinical trials have meant that IP chemotherapy has yet to be universally accepted as a routine first-line therapy. This is likely also due to the perceived complexity of delivery, reported increased toxicity, and development of newer first-line maintenance therapies [36].

Hyperthermic intraperitoneal chemotherapy (HIPEC) involves a single intra-operative administration of heated cytotoxic chemotherapy into the abdomino-pelvic cavity following surgery. The high temperature leads to increased drug penetration and tumour cell sensitivity to cytotoxic chemotherapy and induces apoptosis [40, 41]. First-line HIPEC has been investigated in two clinical trials [42, 43]. In the randomised phase III trial, OVIHIPEC-1, 245 patients diagnosed with FIGO stage III EOC (89.4% HGS) were referred for NACT if IPS was not feasible or would likely have left residual disease of > 1 cm [42•]. Patients were treated with neoadjuvant 3-weekly carboplatin (AUC5/6) and 3-weekly paclitaxel (175 mg/m^2^) for 3 cycles and then randomised intraoperatively to receive HIPEC with IP cisplatin (100 mg/m^2^) or nothing. The cisplatin was administered at the end of cytoreductive surgery after the abdominal cavity had been heated to 40 °C using heated saline. In this trial, HIPEC was associated with a significant improvement in PFS (14.2 versus 10.7 months, HR 0.66, 95% CI 0.50–0.87) and OS (45.7 versus 33.9 months, HR 0.67, 95% CI 0.48–0.94) [42•]. However, the results from OVIHIPEC-1 were inconsistent with those reported in a randomised phase II undertaken in Korea [43]. In this trial, 184 women diagnosed with FIGO stage III–IV EOC were randomised intra-operatively during IPS or DPS, after optimal cytoreduction (RD < 1 cm), to receive HIPEC with IP cisplatin (75 mg/m^2^) or nothing. The abstract reports that there was no difference in PFS or OS between groups (PFS: 20 versus 19 months and OS: 54 versus 51 months), but peer-reviewed publication of this trial is still awaited.

The reason for the inconsistency between studies remains unclear, but the slow recruitment in both trials (9 years in OVIHIPEC-1; 6 years in the Korean study) suggests that HIPEC can only be delivered to a relatively small and select group of patients [44]. In addition, OVIHIPEC-1 was also criticised as the results were not stratified based on prognostic factors such as *BRCA* status and/or histological subtype. These factors could have skewed the data in favour of the HIPEC group, which contained fewer patients with a histological diagnosis associated with a worse prognosis (i.e., mucinous, clear cell or carcinosarcoma). Moreover, the results were also very different between sites, with sites that recruited the most patients reporting worse outcomes in the HIPEC group. The OVIHIPEC-2 trial (NCT03772028) has been designed to address many of the issues that arose in previous trials and to determine if surgery with HIPEC can prolong OS with acceptable morbidity in the context of modern maintenance treatment. Patients that will be recruited are those with FIGO stage III EOC and they will be randomised to receive primary cytoreductive surgery with or without HIPEC with cisplatin.

At present, HIPEC is not widely used as standard first-line treatment and further investigation in randomised phase III trials is necessary [45]. Unfortunately, defining the position of HIPEC and IP chemotherapy in the current era is becoming harder as more effective maintenance therapies and greater understanding of BRCA/HRD start to impact first-line treatment regimens.

## Bevacizumab Maintenance First-Line Therapy

Angiogenesis, the formation of new blood vessels, is a hallmark of cancer [46, 47]. The sensitivity of EOC to vascular endothelial growth factor (VEGF) inhibition is most likely related to the fundamental role that VEGF plays in the physiology of the normal ovary [48]. Indeed, the clinical utility of VEGF inhibition, using the humanised monoclonal anti-VEGF antibody bevacizumab, within first-line treatment of EOC, has been demonstrated in two randomised phase III trials [49, 50].

In ICON7, 1528 women diagnosed with FIGO stage IIB-IV EOC (69% serous adenocarcinoma) were randomised to receive 3-weekly carboplatin (AUC5/6) plus 3-weekly paclitaxel (175 mg/m^2^) with or without 3-weekly bevacizumab (7.5 mg/kg). Bevacizumab was administered concurrently with chemotherapy and continued thereafter for a maximum of 18 cycles in total. The addition of bevacizumab significantly improved median PFS (19.0 versus 17.3 months, HR 0.81, 95% CI 0.70–0.94) [49], but an improvement in median OS was only demonstrated in women considered at “high-risk” of developing relapsed disease (39.7 versus 30.2 months, HR 0.78, 95% CI 0.63–0.97) [51]. High-risk disease included FIGO stage III with > 1 cm of RD following cytoreductive surgery, FIGO stage IV disease and/or inoperable disease [51].

In GOG 218, 1837 patients diagnosed with incompletely resected FIGO stage III or FIGO stage IV EOC (83.6% serous adenocarcinoma) were randomised to receive 3-weekly carboplatin (AUC6) plus 3-weekly paclitaxel (175 mg/m^2^) with or without 3-weekly bevacizumab (15 mg/kg). Bevacizumab was administered concurrently with chemotherapy only (cycles 2–6) or alongside chemotherapy and as maintenance (cycle 2–22) for a maximum of 21 cycles in total. The group of patients that continued bevacizumab as maintenance achieved a significantly improved PFS compared to those that had chemotherapy alone (14.1 versus 10.3 months, HR 0.717, 95% CI 0.0625–0.824) [50]. In keeping with ICON7, GOG 218 also demonstrated that patients with FIGO stage IV disease achieved significantly longer OS (42.8 versus 32.6 months, HR 0.75, 95% CI 0.59–0.95) with bevacizumab [52••].

Following the results of ICON7 and GOG 218, bevacizumab was recommended for use in the first-line management of patients with advanced stage EOC, to be used alongside chemotherapy and continued for 15 (12 in the UK) months as maintenance therapy [5, 6]. It remains unclear if additional cycles of bevacizumab can extend PFS further, and so the results of the BOOST trial (NCT01462890) are eagerly awaited; comparing 15 versus 30 cycle in the first-line setting.

Other anti-angiogenic agents, including nintedanib [53] and pazopanib [54] also showed improved PFS in the first-line setting as maintenance therapies, although these orally administered small molecule tyrosine kinase inhibitors demonstrated an increased incidence of diarrhoea and haematological toxicities.

There has been a global search for biomarkers that could be used to optimise the use of VEGF inhibitors. We have shown that a reduction and subsequent increase in plasma Tie2, in patients with ovarian or colorectal cancer treated with bevacizumab, reflect vascular response and then vascular progression, respectively [55, 56]. Taken in conjunction with similar pharmacodynamic changes in plasma Tie2, induced by cediranib in patients diagnosed with glioma [57], these findings together suggest that plasma Tie2 is the first tumour vascular response biomarker for VEGF inhibitors [58]. Further prospective validation of Tie2 utility is underway in the VALTIVE-1 study.

## Poly(ADP-Ribose) Polymerase Inhibitors

Recently, poly(ADP-ribose) polymerase (PARP) inhibitors have changed the outlook for some women with FIGO stage III/IV HGS carcinoma. Small molecule inhibitors of PARP1 and PARP2 act in *BRCA*-mutant and Homologous recombination-deficient (HRD) tumours, probably through the mechanistic framework of synthetic lethality, although there are several theories for the exact mechanism of action [59]. Approximately 20% of HGS tumours have a germline or somatic *BRCA1/2* mutation and in total up to 50% are HRD [60, 61]; both mechanisms playing important roles in DNA repair and error-free repair of double-strand breaks (DSBs), respectively. In HRD tumour cells, alternative “error-prone” DSB repair pathways (e.g. non-homologous end joining [NHEJ]) are more heavily relied upon, thereby leading to genomic instability [62]. One theory is that PARP inhibitors can exploit this unique molecular feature of HGS carcinoma by inhibiting DNA single-strand break (SSB) repair [63, 64]. Indeed, unrepaired SSBs may form lethal DSB during the G2/S-phase of the cell cycle. Subsequently, HRD tumours that are unable to repair DSBs are more likely to die after exposure to PARP inhibitors. PARP inhibitors prevent repair of SSBs by inhibiting catalytic formation of polymers of ADP-ribose forming and trapping PARP1 on DNA [65, 66]. The NHEJ pathway also appears to play a key role in the way PARP inhibitors work in HRD cells and studies have shown cells with already defective NHEJ may in fact be resistant PARP inhibitors [67, 68].

A number of randomised phase III trials have recently reported the efficacy of PARP inhibitors as first-line maintenance monotherapy [69–71] (Table 3). SOLO-1 reported a 70% reduction in the risk of progression or death in patients with FIGO stage III/IV *BRCA*-mutant (germline or somatic) platinum-sensitive HGS or high-grade endometrioid carcinoma following 24 months of olaparib (300 mg twice daily) compared to placebo (HR 0.30, 95% CI 0.23–0.41) [69••] (Table 3). In the PRIMA trial, 733 patients with FIGO stage III–IV HGS carcinoma were randomised following a complete/partial response to cytoreductive surgery plus platinum-based chemotherapy to receive either niraparib (300 mg once daily, 28-day cycles) or placebo for a maximum of 36 months [70••]. The trial reported an improvement in PFS in all patients with niraparib compared to placebo (13.8 versus 8.2 months, HR 0.62, 95% CI 0.05–0.76). The greatest benefits in PFS were demonstrated in *BRCA*-mutant and/or HRD tumours (Table 3).

**Table 3:** PFS outcomes in PARP inhibitor trials based on genetic stratification [69–71]

PFS (months)	SOLO1		PRIMA		VELIA		PAOLA-1	
	Control arm Placebo	Experimental arm Oral olaparib 300 mg BD maintenance after chemotherapy	Control arm Placebo	Experimental arm Oral niraparib 300 mg OD maintenance after chemotherapy	Control arm Placebo	Experimental arm Oral veliparib 150 mg BD throughout chemotherapy and continued as maintenance	Control arm Placebo + bevacizumab 15 mg/kg	Experimental arm Oral olaparib 300 mg BD maintenance after chemotherapy + bevacizumab 15 mg/kg
Intention-to-treat population	N/A	N/A	8.2	13.8 (HR 0.62, 95% CI 0.50–0.76)	17.3	23.5 (HR 0.68, 95% CI 0.56–0.83)	16.6	22.1 (HR 0.59, 95% CI 0.49–0.72)
All HRD positive (includes BRCA1/2 mutation)	N/A	N/A	10.9	22.1 (HR 0.40, 95% CI 0.27–0.62)	20.5	31.9 (HR 0.57, 95% CI 0.43–0.76)	17.7	37.2 (HR 0.33, 95% CI 0.25–0.45)
HRD positive/*BRCA*-wildtype	N/A	N/A	8.2	19.6 (HR 0.50, 95% CI 0.31–0.83)	N/A	N/A	16.6	28.1 (HR 0.43, 95% CI 0.28–0.66)
*BRCA1/2* mutation	13.8	49.9 (HR 0.30, 95% CI 0.23–0.41)	N/A	N/A	22.0	34.7 (HR 0.44, 95% CI 0.28–0.68)	21.7	37.2 (HR 0.3, 1 95% CI 0.20–0.47)
HR-competent	N/A	N/A	N/A	N/A	N/A	N/A	16.2	16.6 (HR 1.00, 95% CI 0.75–1.35)

In the VELIA trial, 1140 women with FIGO stage III–IV HGSC were randomised prior to receiving first-line multi-modality therapy to one of 3 arms: (A) chemotherapy with concurrent and maintenance veliparib (150 mg twice-daily) or (B) chemotherapy with concurrent veliparib and placebo maintenance therapy or (C) placebo throughout chemotherapy and as maintenance [71••]. For patients randomised to take veliparib with and following chemotherapy, a PFS advantage was demonstrated, again with the greatest benefits reported in patients with *BRCA*-mutant and HRD tumours (Table 3). There was no significant difference in PFS between the group that took veliparib with chemotherapy only in comparison with the placebo group. Interestingly, unlike olaparib or niraparib, veliparib was administered concurrently with platinum therapy, whereas previous trials had shown that combining PARP inhibitors and platinum caused intolerable myelotoxicity [72].

The most common adverse effects of PARP inhibitors reported in trials were myelosuppression, nausea and fatigue (Table 4). The olaparib trial reported fewer incidences of grade 3 or above anaemia or neutropenia than niraparib and veliparib. More rare, but clinically significant, adverse effects such as pneumonitis, myelodysplastic syndrome and acute myeloid leukaemia were reported infrequently in the trials but more commonly with olaparib (Table 4) [69–71].

**Table 4:** Common and serious adverse events reported in PARP inhibitor trials [69–71]

	SOLO-1 trial—olaparib group		PRIMA trial—niraparib group		VELIA trial—veliparib throughout group	
Event	Any grade (% of patients)	Grade 3 or 4 (% of patients)	Any grade (% of patients)	Grade 3 or 4 (% of patients)	Any grade (% of patients)	Grade 3 or 4 (% of patients)
Anaemia	39	22	63	31	64	38
Neutropenia	23	9	26	12	75	58
Thrombocytopenia	11	1	46	29	58	28
Nausea	77	1	57	1	80	8
Fatigue	63	4	35	2	69	8
Pneumonitis	2	N/A	0	N/A	0	N/A
Acute myeloid leukaemia	1	N/A	0	N/A	0.3	N/A
Myelodysplatic syndrome	0	N/A	0.2	N/A	0*	N/A

*There was an incidence of myelodysplastic syndrome in 1 patient (0.2%) in veliparib combination only group in this trial [71]

In PRIMA and VELIA, HRD tumours were identified through the myChoice® companion diagnostic (CDx) test (Myriad Genetics, Inc., Salt Lake City, UT, USA). This assay determines an HRD “score” using three algorithms calculating genetic aberrations in single nucleotide polymorphisms (SNPs). These algorithms assess genetic characteristics of tumour DNA, including loss of heterozygosity (LOH), telomeric allelic imbalance (TAI) and large-scale transitions (LST), all surrogate markers of genomic instability [73–75]. The assay provides an HRD “score” that is calculated based on the accumulative score of each algorithm (LOH, TAI and LST). A tumour is considered HRD according to a set score e.g. in PRIMA HRD tumours had a HRD score ≥ 42, whereas in VELIA, the threshold was set at 33 [70, 71]. In both trials, women with HRD tumours who received the PARP inhibitor had significantly longer PFS compared to those who received placebo. The difference in the HRD score may impact upon the choice of PARP inhibitor used in front-line therapy.

Overall, the outcomes of these trials show that patients with known *BRCA1/2* mutations benefit from PARP inhibitors as maintenance monotherapy after a response to first-line platinum-based chemotherapy. In addition, those patients with HRD tumours benefit from niraparib or veliparib, and have similar risks of adverse effects (Table 4). Although further analysis of OS is awaited in all three trials, surrogate markers of OS including time-to-first subsequent therapy (TFST), progression-free survival 2 (PFS2) and time-to-second subsequent therapy (TSST) suggest early indications of long-term survival benefit [69••]. It is becoming increasingly important to test all patients for germline and somatic *BRCA1/2* mutations at diagnosis to help guide maintenance options, and recent approval in the USA of the Myriad myChoice® CDx represents an additional biomarker for assessment in *BRCA* wildtype patients.

## Immunotherapy

Immune checkpoint blockade with anti-CTLA-4 and/or anti-PD-1/PD-L1 inhibitors is not routinely used in the management of HGS carcinoma at present. Early phase trials have reported relatively disappointing response rates of between 10 and 20% [76–78]. Indeed, the randomised first-line phase III trial, JAVELIN Ovarian 100 trial (NCT02718417) was prematurely discontinued due to insufficient activity. This trial had been recruiting patients with stage III–IV EOC that were due to start platinum-based chemotherapy. Patients were to be randomised to receive 3-weekly avelumab (an anti-PD-L1 antibody) during chemotherapy or during chemotherapy and as maintenance therapy or chemotherapy alone. As a result of the interim results of JAVELIN Ovarian 100, another randomised phase III trial, JAVELIN Ovarian PARP 100 trial (NCT03642132), investigating avelumab plus the PARP inhibitor, talazoparib, was also prematurely discontinued [79].

*BRCA*-mutated HGS carcinoma and *TP53*-mutated EOC often contain increased number of tumour-infiltrating lymphocytes and express PD-1/PD-L1 [80, 81], suggesting that immune checkpoint inhibitors should be effective. However, copy number variation is more common than neoantigen formation in HGS and hence checkpoint inhibitors used alone have not yielded meaningful activity. In an attempt to overcome this problem, multiple trials have been initiated that aim to boost the immune response through concurrent administration of immune oncology agents with PARP and/or VEGF inhibitors (Table 5).

**Table 5:** Ongoing first-line phase III clinical trials involving immune checkpoint inhibitors with combination regimens

Trial name	GOG3015/ENGOT OV39	FIRST trial/ENGOT Ov-44	DUO-O/ENGOT Ov-46	ENGOT Ov-43/MK7339-001	ATHENA
Trial identifier	NCT03038100	NCT03602859	NCT03737643	NCT03740165	NCT03522246
Histology	Epithelial	Epithelial	Epithelial	Epithelial	Epithelial
FIGO stage	III–IV	III–IV	III–IV	III–IV	III–IV
ECOG PS	0–2	0–1	0–2	0–1	0–2
Investigational drugs	Atezolizumab	Dorstarlimab Niraparib	Durvalumab Olaparib	Pembrolizumab Olaparib	Nivolumab Rucaparib
Control arm-chemotherapy phase	IV paclitaxel + IV carboplatin + IV bevacizumab + IV atezolizumab placebo	Arm 1: Standard of care chemotherapy + IV dorstarlimab placebo	Arm 1: IV paclitaxel + IV carboplatin + IV bevacizumab + IV durvalumab placebo	Arm 3: IV paclitaxel + IV carboplatin ± IV bevacizumab + IV pembrolizumab placebo	N/A
Control arm-maintenance phase	IV bevacizumab + IV atezolizumab placebo	Arm 1: IV dorstarlimab placebo + PO niraparib placebo	Arm 1: IV bevacizumab + IV durvalumab placebo + PO olaparib placebo	Arm 3: ± IV bevacizumab + IV pembrolizumab placebo + PO olaparib placebo	Arm D: IV nivolumab placebo + PO rucaparib placebo
Experimental arm (1)-chemotherapy phase	IV paclitaxel + IV carboplatin + IV bevacizumab + IV atezolizumab (1200 mg, three-weekly)	Arm 2: Standard of care chemotherapy + IV dorstarlimab placebo	Arm 2: IV paclitaxel + IV carboplatin + IV bevacizumab + IV durvalumab	Arm 1: IV paclitaxel + IV carboplatin ± IV bevacizumab + IV pembrolizumab (200 mg, three weekly)	N/A
Experimental arm (1)-maintenance phase	IV bevacizumab + IV atezolizumab (1200 mg, three-weekly)	Arm 2: IV dorstarlimab placebo + PO niraparib	Arm 2: IV bevacizumab + IV durvalumab + PO olaparib placebo	Arm 1: ± IV bevacizumab + IV pembrolizumab (200 mg, three-weekly) + PO olaparib (300 mg, twice-daily)	Arm A: IV nivolumab (four-weekly) + PO rucaparib (twice-daily)
Experimental arm (2)-chemotherapy phase	N/A	Arm 3: Standard of care chemotherapy + IV dorstarlimab	Arm 3/patients with tBRCAm: IV paclitaxel + IV carboplatin ± IV cevacizumab + IV durvalumab	Arm 2: IV paclitaxel + IV carboplatin ± IV bevacizumab + IV pembrolizumab	N/A
Experimental Arm (2)-maintenance phase	N/A	Arm 3: IV dorstarlimab + PO niraparib	Arm 3/patients with tBRCAm: ± IV bevacizumab + IV durvalumab + PO olaparib	Arm 2: ± IV bevacizumab + IV pembrolizumab (200 mg, three-weekly) + PO olaparib placebo	Arm B: IV nivolumab placebo + PO rucaparib (twice-daily)
Experimental arm (3)-maintenance phase	N/A	N/A	N/A	N/A	Arm C: IV nivolumab (four-weekly) + PO rucaparib placebo
Primary outcome	PFS in intention to treat (ITT) population PFS in programmed cell death-ligand 1 (PD-L1)-positive subpopulation OS in ITT population OS in PD-L1-positive population	PFS	PFS–in non-tBRCAm patients	PFS OS	PFS

## Combination First-Line Maintenance Treatment

Recent clinical trials in EOC have started to evaluate combinations of VEGF and PARP inhibitors. An initial randomised phase II treatment trial described increased PFS and OS in patients with recurrent platinum-sensitive EOC that were treated with a combination of cediranib (30 mg, once daily) plus olaparib (capsules; 200 mg, twice-daily) compared to olaparib alone. Importantly, this benefit was more evident in patients with no *BRCA1/2* mutation [82, 83]. Mechanistic studies have shown that the increased activity of combination therapy is related to hypoxia-driven reduction in the proteins involved in HR repair, which then resulted in reduced BRCA expression and function, sensitising *BRCA1/2* wild-type cells to PARP inhibitors [84].

The only trial to assess the combination of PARP and VEGF inhibition in first-line management of EOC is PAOLA-1 [85••]. In this randomised phase III trial, 806 women with FIGO stage III–IV high-grade serous or endometrioid ovarian cancers (95.8% HGS) had responded to first-line platinum-taxane chemotherapy, and bevacizumab were randomised to receive either ongoing 3-weekly bevacizumab (15 mg/kg) as maintenance for a maximum of 15 months total with olaparib (300 mg, twice-daily) for up to 24 months, or bevacizumab with placebo. Tumour samples were analysed for *BRCA* mutation and HRD testing prior to randomisation, with an HRD score of ≥ 42 indicating a positive result. A PFS benefit was demonstrated in the combination arm (investigator-assessed PFS: 22.1 versus 16.6 months, HR 0.59, 95% CI 0.49–0.72), with the most profound PFS advantage being reported in patients with BRCA1/2 mutations (Table 3) [85••]. The main criticism of PAOLA-1 was that there was no single agent olaparib maintenance monotherapy arm included, which made it difficult to determine if the PFS advantage in patients with HRD tumours was due to synergy between bevacizumab-olaparib or olaparib alone.

Results from phase III trials combining immune checkpoint inhibitors with VEGF or PARP inhibitors in first-line management of EOC are eagerly awaited (Table 5). The rationale for use of PARP inhibitors is the increased DNA damage in cells, which might increase neoantigen formation and hence the effects of immunotherapy [86]. In contrast, VEGF inhibitors act by converting an immunosuppressive tumour microenvironment (TME) to an immunosupportive one, potentially increasing sensitivity to immunotherapy [87, 88]. If the results of combinational agents prove effective, it will be critical to develop biomarkers that will optimise treatment of the right patients so that cost-effectiveness is delivered.

## Conclusion

The first-line treatment of HGS carcinoma now builds on the well-established backbone of surgery and paclitaxel/platinum-based chemotherapy. Surgery can be carried out upon diagnosis or after NACT. The role of IP chemotherapy and HIPEC remains undefined.

Maintenance monotherapies with bevacizumab and PARP inhibitors have demonstrated progression-free survival benefits in certain clinically and molecularly defined subgroups. Key questions remain over the benefits of combination regimens and whether molecularly targeted biomarkers can optimise patient stratification. Nevertheless, 70% of patients are likely to develop recurrent disease and require further treatment. Bevacizumab has proven effective in the re-use setting [89] but trials on the re-use of PARP inhibitors are currently ongoing (NCT03136987). Therefore, based on current evidence, it is vital to decide which drugs should be used upfront and which should be saved for use in relapse.

Recent phase III trials highlight the future need for randomised trials on the re-use of maintenance therapies, the upfront testing of BRCA and HRD status and the ongoing development of biomarkers for use of VEGFi.
